# Forestier's disease presenting with dysphagia and disphonia

**DOI:** 10.11604/pamj.2014.17.168.2453

**Published:** 2014-03-06

**Authors:** Jaafar Najib, Stephane Goutagny, Mathieu Peyre, Thierry Faillot, Michel Kalamarides

**Affiliations:** 1 Service de Neurochirurgie, CHU Beaujon, Paris, France

**Keywords:** Forestier's disease, dysphagia, disphonia

## Abstract

Forestier's disease, also known as diffuse idiopathic skeletal hyperostosis (DISH), is a pathology of vertebral bodies characterised by exuberant osteophytis formation. Forestier's disease is usually managed conservatively. Surgical resection of the osteophytes is reported to be an effective treatment for severe cases and/ or cases with airway obstruction. We report a 55-year-old man presenting with 6 months’ progressive dysphagia and dysphonia. He was managed successfully with an anterior cervical osteophytectomy without fusion. A literature review is included.

## Introduction

Diffuse idiopathic skeletal hyperostosis (DISH) is an ossifying diathesis characterized by spinal and peripheral enthesopathy. It was first described as senile ankylosing hyperostosis of the spine by Forestier and Rotes-Querol in 1950. In the 1970s, Resnick et al coined the term DISH for this systemic entity.

## Patient and observation

**Figure 1:**
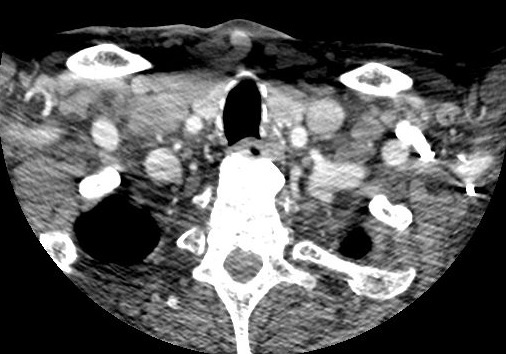
Neck computed tomographic scan showed osteoarthritic changes consistent of anterior cervical osteophytes causing displacement of the upper airway and compression of esophagus

A 78-year-old man with obstructive sleep apnea syndrome, peripheral vascular disease, arterial hypertension, surgery for lumbar spinal stenosis in 2008 and cervical spondylotic myelopathy, presented with a 6-month history of progressive dysphagia, dysphonia and alteration of general state. The patient underwent a percutaneous endoscopic gastrostomy. On examination, he was neurologically intact with no clinical features of cervical spondylosis. Neck computed tomography showed anterior cervical osteophytes displacing the upper airway and compressing the esophagus (Figure 1).

**Figure 2:**
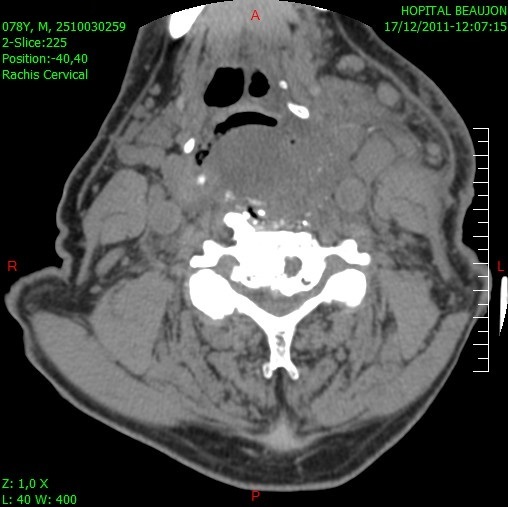
Postoperative sagittal cervical CT scan with bone windowing showing complete resection of anterior osteophytes

A standard anterior cervical approach was undertaken via a transverse skin incision. Going anterior to the carotid sheath, we identified the palpable osteophytes and dissected the longus coli muscle laterally using cutting diathermy. Guided by serial intraoperative fluoroscopy, we drilled away the osteophytes, obtaining a normal contour to the anterior aspect of the C4 to C6 vertebral bodies. The cut bone was then drilled using a diamond burr. The procedure was uncomplicated and he was discharged the following day with rapid resolution of his dysphagia. A check cervical CT scan was performed (Figure 2) prior to his discharge. At 8 weeks of follow-up in clinic, he remained well.

## Discussion

Foretier's disease, also known as diffuse idiopathic skeletal hyperostosis (DISH), is an ossifying diasthesis characterized by spinal and peripheral enthesopathy. It was first described as senil ankylosing hyperostosis of the spine by Forestier and Rothe-Querol in 1950 [1]. In the 1970, Resnick et al [2, 3] coined the term DISH for this systemic entity. They further advocated following three diagnostic criteria: (a) the presence of following ossification of the anterior longitudinal ligament (OALL) of at least four contiguous vertebral bodies, (b) the preservation of intervertebral disc height, and (c) the absence of apophyseal joint bone ankylosis and sacroiliac joint erosion, intra articular osseous fusion or sclerosis.

This disease most commonly affects the paraspinal ligaments, predominatly the anterior longitudinal ligament and occasionally the posterior longitudinal ligament [4, 5]. The thoracic region is almost always involed (96%). The lumbar (90%) and cervical regions (78%) are affected to a lesser extent [4, 6, 7]. At times, soft tissue thickening and calcification can olso occur at peripheral joints, particularly the femur, patella or the metatarsal joint [4, 8, 9].

There is a male predominance of Forestier's disease, mainly affecting elder individuals in their fifth or sixth decades [4, 9, 10]. Forestier's disease is reported to affect 1 in 4 males, and aproximately 1 in 7 females, over the age of 50 years [6]. In 1926, Moshe [11] was the first to report dysphagia secondary to cervical osteophytes. Clinical studies have shown that 17- 28% of patients with DISH manifested symptoms of dyphagia due to cervical osteophytes [12]. Large osteophytes do cause swalowing disorders through a variety of mechanisms, including: (a) direct mechanical compression of the pharynx and oesophagus [2], (b) disturbances of normal epiglottis tilt over the laryngeal inled by the osteophytes at C3-C4 level [13, 14]. (c) Inflammatory reactions in the tissues arround the oesophagus [15, 16], and crico-pharyngeal spasm [1].

As the terminology of the disease suggests, the pathogenesis is unknown [6]. However, recent research have etablished that obesity and a first degree relative with hypertension or diabetes mellitus are significal risks factors for developing Forestier's disease [17, 18]. Conservative treatment has been indicated for the initial management of the most patients [1, 16, 19, 20]. Surgical resection of the osteophytes has been reported to be an effective treatment for severe cases and/ or cases with airway obstruction [14, 21, 22].

Many surgical reports about DISH related dysphagia have been described in the literature [16, 23]: however, few of these include postsurgical results for more than 2 years. Little study has been given to the regrowth of osteophytes after surgical resection. Hirano et al [24] reported that two patients developed asymptomatic reccurrent osteophytic formation at the operated site 4,5 years after surgical resection. In Kei Miyamato et al study, the mean postoperative increase of size of the larget reccurent osteophyte in each patient was about 1mm/year. It seems possible that most patients will return to their preoperative condition 14-20 years after surgery because the size of the largest reccurent osteophyte will reach 14-20 mm, wich is equal to the preoperative size [25].

## Conclusion

Although most patients with Forestier's disease can be managed conservatively, for patients with symptoms justifying intervention, surgery is a safe and effective option. Patients do, however, require long-term follow-up.
